# Image-Guided Surgical Robotic System for Percutaneous Reduction of Joint Fractures

**DOI:** 10.1007/s10439-017-1901-x

**Published:** 2017-08-16

**Authors:** Giulio Dagnino, Ioannis Georgilas, Samir Morad, Peter Gibbons, Payam Tarassoli, Roger Atkins, Sanja Dogramadzi

**Affiliations:** 1 Bristol Robotics Laboratory, University of the West of England, Coldharbour Lane, Bristol, BS161QY UK; 2 Aston University, Birmingham, B47ET UK; 3 University Hospitals Bristol, Upper Maudlin Street, Bristol, BS28HW UK

**Keywords:** Computer-assisted surgery, Medical robotics, Percutaneous fracture surgery, Navigation, Virtual planning, Cadaveric experimental study

## Abstract

Complex joint fractures often require an open surgical procedure, which is associated with extensive soft tissue damages and longer hospitalization and rehabilitation time. Percutaneous techniques can potentially mitigate these risks but their application to joint fractures is limited by the current sub-optimal 2D intra-operative imaging (fluoroscopy) and by the high forces involved in the fragment manipulation (due to the presence of soft tissue, e.g., muscles) which might result in fracture malreduction. Integration of robotic assistance and 3D image guidance can potentially overcome these issues. The authors propose an image-guided surgical robotic system for the percutaneous treatment of knee joint fractures, i.e., the robot-assisted fracture surgery (RAFS) system. It allows simultaneous manipulation of two bone fragments, safer robot-bone fixation system, and a traction performing robotic manipulator. This system has led to a novel clinical workflow and has been tested both in laboratory and in clinically relevant cadaveric trials. The RAFS system was tested on 9 cadaver specimens and was able to reduce 7 out of 9 distal femur fractures (T- and Y-shape 33-C1) with acceptable accuracy (≈1 mm, ≈5°), demonstrating its applicability to fix knee joint fractures. This study paved the way to develop novel technologies for percutaneous treatment of complex fractures including hip, ankle, and shoulder, thus representing a step toward minimally-invasive fracture surgeries.

**Electronic supplementary material:**

The online version of this article (doi:10.1007/s10439-017-1901-x) contains supplementary material, which is available to authorized users.

## Introduction

Traumatic fractures can lead to devastating clinical consequences for patients and substantial burden to healthcare systems. It is estimated that impaired healing will occur in 5–10% of the 5.6 million fractures that occur annually only in the United States, and 10% of those would require a second surgery to heal.^23^ Joint fractures require 180,000 surgical procedures annually in the United Kingdom, creating £3.7 billion cost to the National Health System (NHS).^25^ To avoid painful arthritis and/or additional surgeries, the fragments must be correctly aligned and fixed.^29^ This often involves an open incision (i.e., open surgery) to expose the fractured bones and allow the surgeon to perform the anatomical reduction, i.e., to reposition and align the fragments as precisely as possible, ensuring the correct joint functionality. Once reduced, the fracture is fixed using plates and screws or intramedullary nails.^12^ Open procedures are associated with extensive soft tissue damage, higher risk of infection, longer hospitalization and rehabilitation time, and higher costs.^21^ Percutaneous techniques allow the surgeon to manipulate the fracture fragments through small incisions in the flesh, thereby gaining advantages of minimally invasive surgical approach.^14^ However, these techniques are limited by static two-dimensional (2D) intra-operative fluoroscopic imaging often inadequate for the three-dimensional (3D) fragment alignments. The 2D field of view does not provide enough information to the surgeon regarding the fracture alignment and rotation, necessitating multiple intra-operative images. This leads to prolonged radiation exposure of patient and staff^1^ or, in up to 5% of cases, expensive revision operations to correct mal-positioned fractures.^23^ The problem is particularly evident when dealing with intra-articular fractures that involve a joint space, e.g., a distal femur or proximal tibia, where optimal anatomical reduction and pose correction of the articular surface is a 3D problem, typically difficult to resolve using 2D imaging.^13^ Moreover, the high forces between bone fragments and muscular attachments during the reduction process often prevent correct reduction movements and occasionally result in suboptimal fracture reduction.^28^ Deforming forces from muscular attachments—which must be counteracted during the reduction process—cause characteristic displacement patterns. The gastrocnemius typically causes a hyperextension deformity of the distal femoral articular block, i.e., this flexes the distal fragment, causing posterior displacement and angulation; the quadriceps and hamstrings exert proximal traction, resulting in shortening of the lower extremity.^12^ Varus angulation may also result at the fracture site from the pull of the adductor muscles. In more severe fractures where there is intercondylar involvement, rotational deformity may also occur further increasing the complexity of the reduction maneuver.^21^

Integration of robotic manipulation and 3D image guidance can increase reduction accuracy when using the minimally invasive access to the fracture fragments. In image-guided procedures, the surgeon is guided by images from different modalities, including CT and fluoroscopy, allowing the surgery to be performed using a much smaller incision than in traditional open surgery. Robotic tools might be used together with image-guidance to perform minimally invasive surgery. As the surgeon watches the images on the screen, they actuate the robot using a dedicated controller. For surgeons, image-guided interventions using robots also have the advantage of reducing fatigue during long or tiring operations, such as in orthopedic surgery.

In the last few decades, robotic surgical systems with 3D image guidance have been proposed^2^,^17^,^20^,^31^,^33^,^35^,^36^ to improve fracture surgeries. Buschbaum *et al*.^2^ and Warisawa *et al*.^35^ developed systems for computer-assisted repositioning of femoral fractures using 3D-CT images. Joskowicz *et al*.^20^ presented FRACAS, a computer-aided system providing image-guidance to the surgeon to reduce a long bone fracture. Westphal *et al*.^36^ reported a robotic system for the reduction of femur shaft fractures based on a telemanipulated industrial serial robot and 3D imaging data generated by intra-operative 3D fluoroscope. Tang *et al*.,^31^ Graham *et al*.,^17^ and Wang *et al*.^33^ utilized a parallel robot for the reduction in diaphyseal femur fractures based on 3D CT image reconstruction process for pre-operative planning. The application of the above systems is restricted to long bone fractures, which usually have a smaller number of larger fragments that can be managed using the current 2D imaging. Joint fractures typically require higher reduction accuracy to restore the articular surface 21 (3D problem) and therefore are more difficult to solve using 2D imaging. To the best of our knowledge, no image-guided robotic systems for the reduction of joint fractures have been reported in the relevant literature.

Earlier research by the authors of this paper towards improving percutaneous reduction of intra-articular fractures^9^,^27^ has resulted in the creation of an image-guided robotic system prototype.^7^ Image-guidance and robotic assistance are combined to help the surgeon to achieve accurate reduction of distal femur fractures (DFF) with minimum damage to soft tissue. More in detail, CT images of a DFF are acquired pre-operatively and processed to generate 3D models of the fracture. Such models are imported into the reduction software which allows the surgeon to pre-plan the reduction of the fracture, by virtually manipulating 3D models (virtual reduction). Motion commands for the robotic system are generated based on the virtual reduction and the bone fragments connected to robotic manipulator are repositioned accordingly, achieving the physical reduction of the fracture. Validation trials on bone phantoms proved that the prototype can successfully reduce a 1-fragment DFF with a reduction accuracy of 1.15 mm, 1.3°.^7^ These trials exposed limitations that might restrict the system’s clinical use. (1) *Simultaneous manipulation of 2 fragments* the earlier prototype could manipulate only one fragment at the time. Complex DFFs are multi-fragmented and require higher surgical skills to be reduced (e.g., 33-C1, see Figs. 5a, 5b).^12^,^21^ (2) *Rigid Robot*-*Bone Attachment* in the previous prototype,^7^ the robot end-effector was attached to the bone through a commercial orthopedic pin, screwed into the fragment. The prototype was tested on bone phantoms (Sawbones) with the orthopedic pin glued to the bone preventing rotation of the pin inside the fragment during robotic manipulation. Clearly, this cannot be done in a clinical procedure. The soft tissue connected to the fracture fragments creates reactive forces to the reduction force applied to the fragment through the manipulating pin. If the pin is not rigidly connected to the robot end-effector and the fragment, the reduction force would not translate to the fragment, potentially compromising the reduction procedure. (3) *Traction capability* the soft tissue in the knee joint (i.e., ligaments, tendons, cartilage, muscles) present in multi-fragmented DFFs require traction of the tibia to restore the original length and rotation of the joint.^12^ The traction also creates the space inside the knee joint required for the manipulation of the fragments. Traction capability was not included in the earlier prototype. (4) *Clinical Workflow* the clinical workflow for the reduction of joint fractures using the earlier RAFS prototype^7^ presented two main issues: (i) The pins were inserted into the bones outside the operating theatre which is typically done in the operating theatre; (ii) The pins should be inserted into the fragments *after* getting the CT scan of the fracture, allowing for full pre-operative planning, and reducing the surgical time. (5) *Reliable System Evaluation:* Presence of the soft tissue plays a key role in the reduction process.^18^ Although the earlier prototype was successfully tested in laboratory on knee phantoms with simulated soft tissue,^7^,^9^ RAFS system needs further evaluation on cadaver tissue specimens to better understand interaction between real anatomical structures and the robotic system.

This paper significantly extends the prior work carried out by this paper’s authors allowing the simultaneous manipulation of two fragments, introducing a novel robot-bone attachment system, including a traction robot, designing a new clinical workflow, and testing the usability of the complete RAFS system on clinically relevant trials performed on cadaver samples.

## Materials and Methods

### RAFS Surgical System

The key requirements for the new RAFS system are: (1) manipulate 2 bone fragments at the same time with a clinically acceptable reduction accuracy, i.e., ≈1 mm (translational), ≈5° (rotational)^7^,^21^; (2) percutaneously attach and manipulate bone fragments, minimizing soft tissue damage; (3) apply traction force to the foot to extend the joint; and (4) provide pre-operative planning and real-time intra-operative 3D imaging to visualize the three-dimensional fracture configuration.

**Figure 1:**
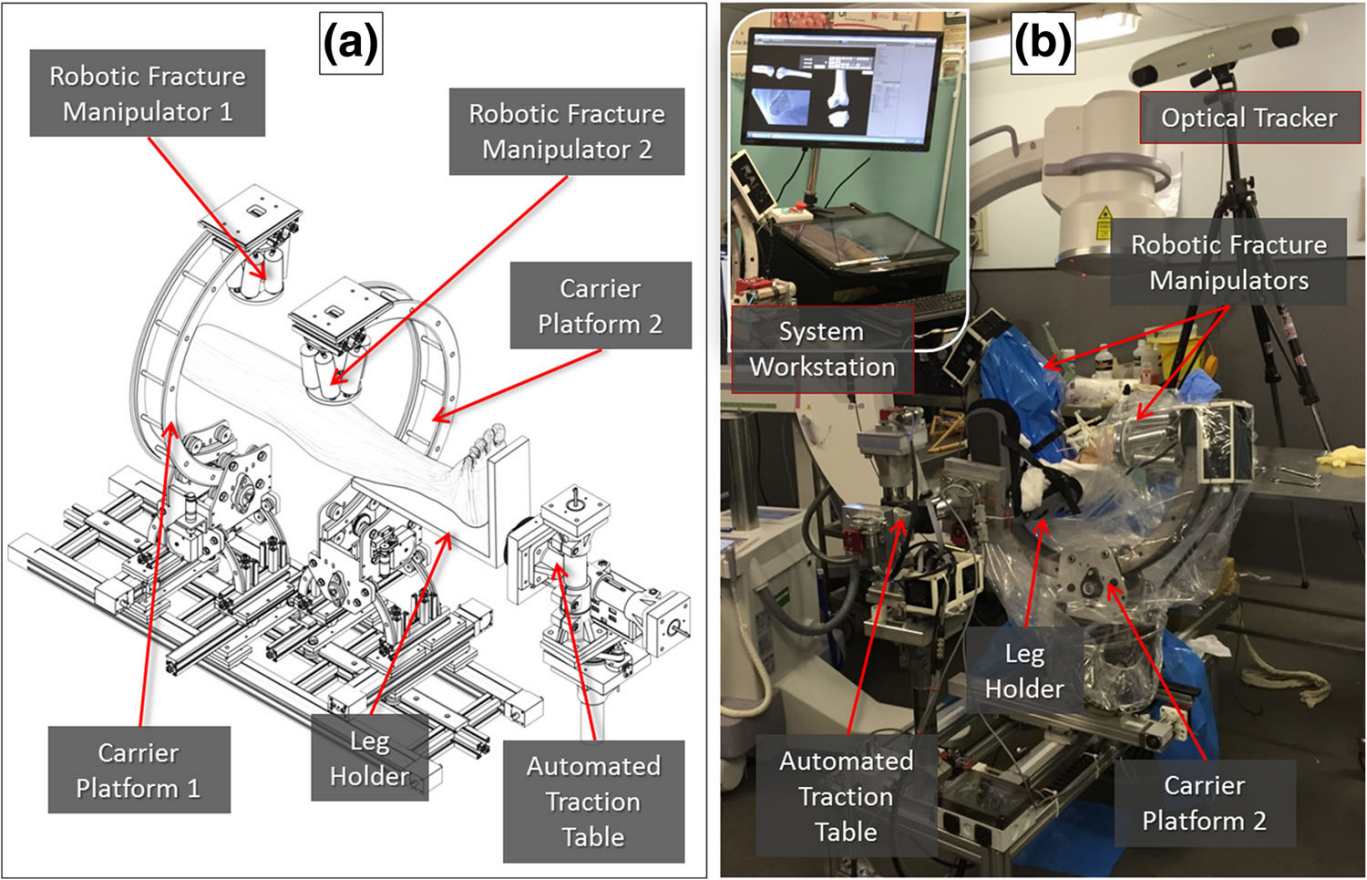
The RAFS surgical system: schematics of the robotic system (a) and its integration with the navigation system in the cadaver laboratory (b).

The new RAFS surgical system—shown in Fig. 1—is based on two Robotic Fracture Manipulators (RFM1, RFM2), i.e., computer-controlled 6DOF parallel-robots with 6DOF load cells enabling force control. Each RFM is connected to a bone fragment through a custom-designed manipulation pin (described below) for fragment manipulation (0.03 ± 0.01 mm translational accuracy and 0.12 ± 0.01° rotational accuracy^11^). Each RFM is mounted on a carrier platform (CP1, CP2) (4-DOF, computer-controlled), which is used for positioning the RFM close to the manipulation pin. The implementation of two RFM-CP systems allows the simultaneous manipulation of two fragments. Kinematics analysis of RFM and CP is reported in Supplement S0 and fully described in Ref. 7.

**Figure 2:**
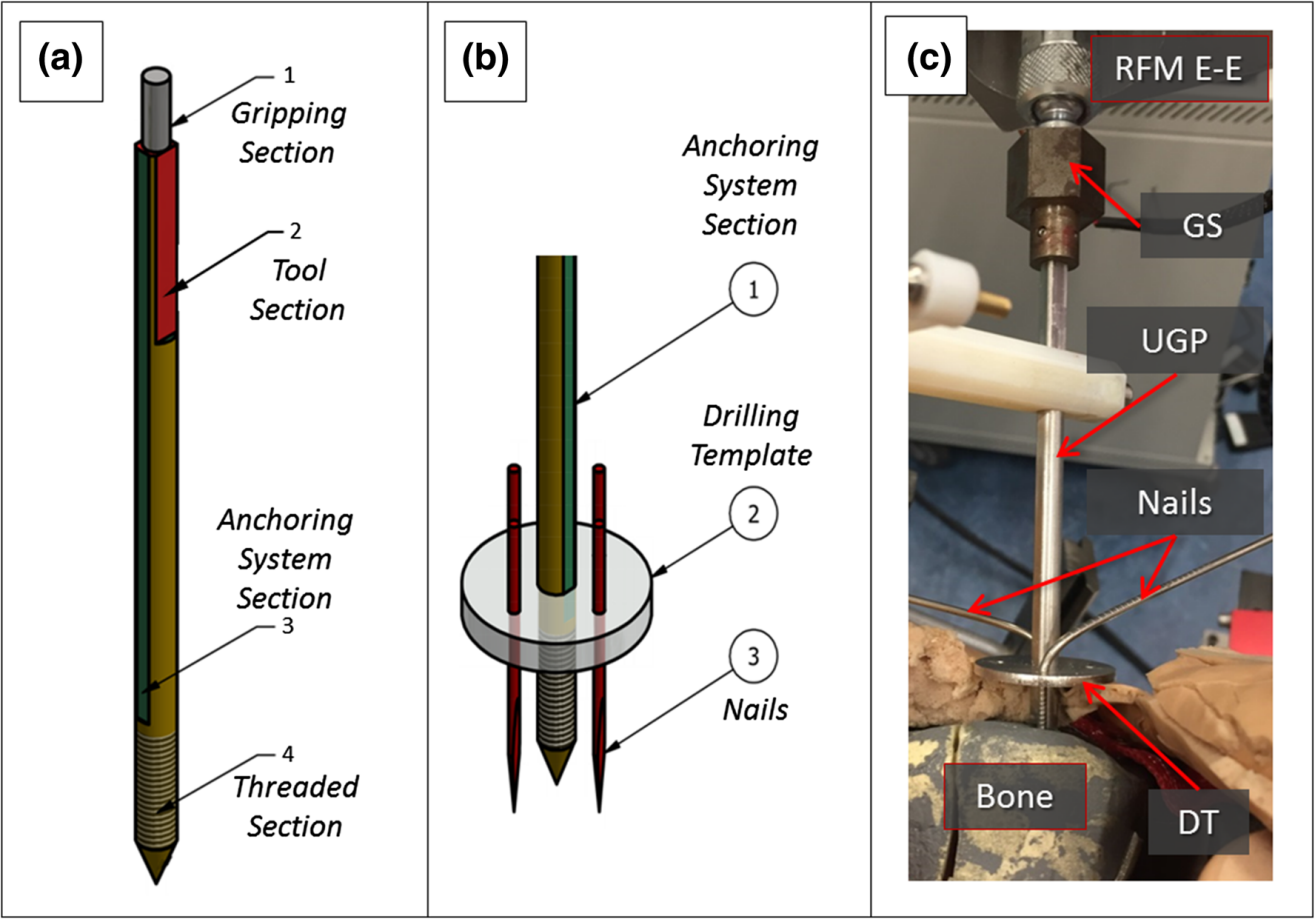
Robot-bone attachment system: CAD drawings of the Unique Geometry Pin (UGP) (a) and the anchoring system (AS) (b). The UGP is secured in the Gripping System (GS) and securely interconnects the RFM end-effector with the bone fragment (c).

A gripping device was designed to securely connect the RFM end-effector and the bone fragment. It consists of a Unique Geometry manipulation Pin (UGP), an Anchoring System (AS), and a Gripping System (GS) (Fig. 2). The UGP (Fig. 2a) is a custom-designed orthopedic manipulation pin [6 mm diameter (D), 142 mm length (L)]. It has 4 parts: (1) *gripping section* (cylinder, D = 4 mm, L = 12 mm) to be connected to the RFM end-effector; (2) *tool section* (L = 33 mm), three-flat-faces unique geometry to which a tool (registration tool or optical tool, see below) can be mounted in a unique orientation, enabling the 3D imaging system^7^,^9^; (3) *anchoring system section* (L = 67 mm), two-flat-faces geometry on which the AS is fixed. This geometry prevents the AS to rotate around the UGP; (4) *threaded section* (L = 30 mm), a M6 × 1 metrical thread screwed into the bone fragment by the surgeon. The AS (Fig. 2b) is a custom designed system that firmly embeds the UGP into the bone fragment using a drilling template (DT) to hold four stainless steel nails. The surgeon drills a UGP into the bone fragment, slides the DT over and drills the 4 nails into the bone fragment through the holes on the DT. The AS assures that the UGP is securely connected to the bone fragment. The GS (Fig. 2c) is mounted on the RFM end-effector and consists of an adjustable spherical joint that can freely orient a specially designed insert. The insert can fit in the gripping section of the UGP. The gripping section of the UGP is connected to the RFM end-effector through the GS and locked with 4 grub screws. This configuration ensures that the force/torque applied by the RFM is fully transferred to the bone fragment to achieve the desired anatomical reduction.

**Figure 3:**
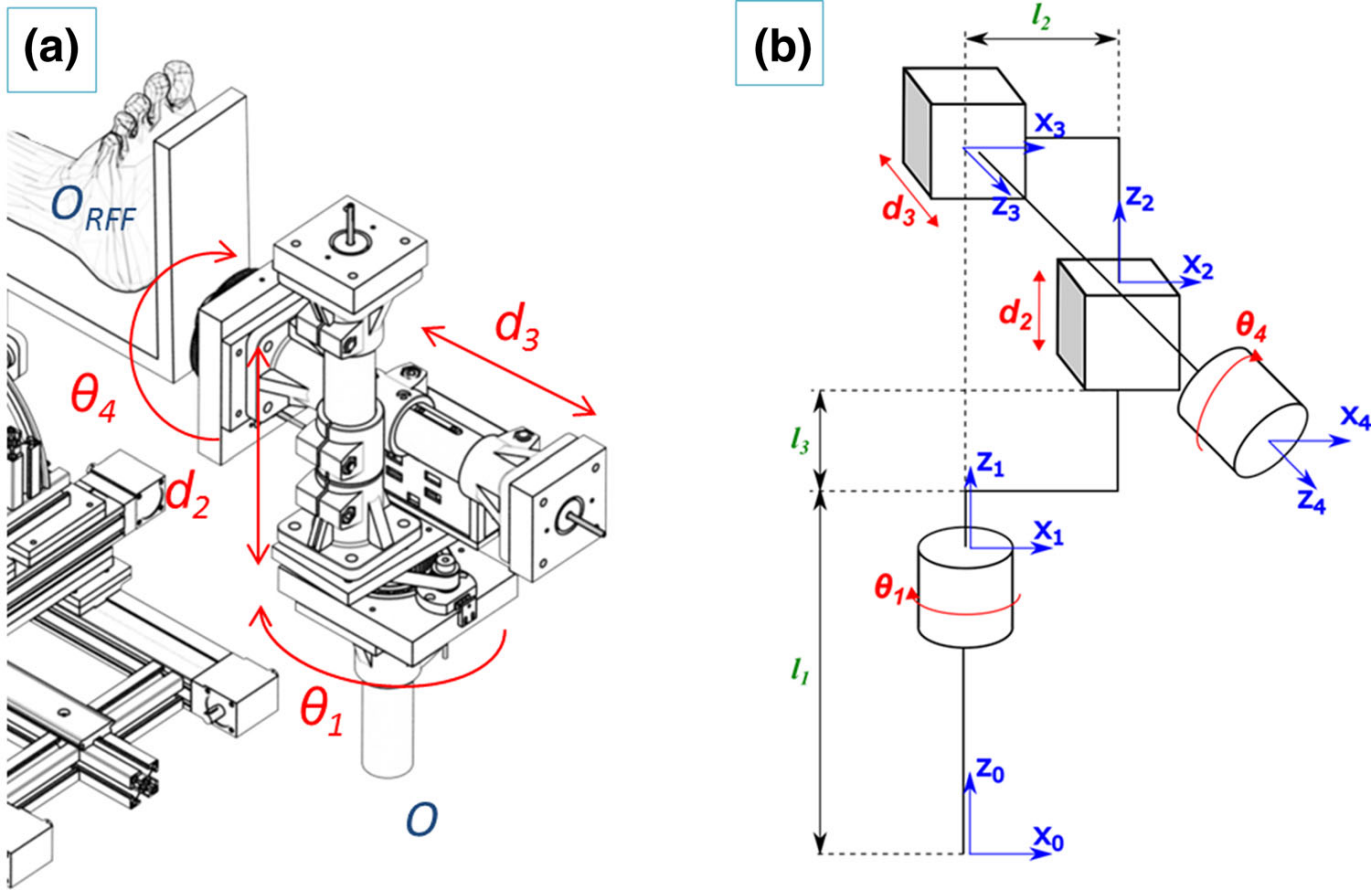
ATT kinematics: rotational (*ϑ*_1_, *ϑ*_4_) and prismatic (*d*_2_, *d*_3_) DOF (a), and DH kinematics chain (b).

As mentioned above, DFF requires traction of the tibia to restore the original length and rotation of the joint.^12^ In the current clinical practice, this is performed by pulling the patient’s foot manually or using a traction table. This allows the surgeon to apply a constant and adjustable traction force to facilitate the reduction process. A computer-controlled version of the traction table, i.e., the automated traction table (ATT) has been introduced in the RAFS system. The ATT is a 4-DOF mechanism, (two prismatic and two revolute joints) (Fig. 3), connected to the tibia through an orthopedic boot and a leg holder. A 6-DOF load cell is mounted between the ATT and the boot holding the patient’s foot to monitor the traction force and torque. The forward kinematics of the ATT is based on the Denavit-Hartenberg (DH) analysis (Fig. 3). The DH parameters are defined using the joint vector:

1 $$\varvec{q} = \left[ {\theta_{1} d_{2} d_{3} \theta_{4} } \right]$$

where, *θ*_1_ is the rotation of the ATT around the axis perpendicular to the limb, *d*_2_ is a displacement perpendicular to the limb axis, *d*_3_ is a displacement along the limb axis, and *θ*_4_ is the rotation around the limb axis. The transformation matrix between the ATT origin {*O*} and the base of the foot reference frame {*O*_RFF_} is given by:

2 $$^{O} \varvec{T}_{{O_{\text{RFF}} }} = \left[ {\begin{array}{*{20}c} {c_{1} c_{4} } & { - c_{1} s_{4} } & { - s_{1} } & { - d_{3} s_{1} } \\ {s_{1} c_{4} } & { - s_{1} s_{4} } & { c_{1} } & { d_{3} s_{1} } \\ { - s_{4} } & { - c_{4} } & { 0 } & { d_{2} + l_{1} + l_{3} } \\ 0 & 0 & 0 & {1 } \\ \end{array} } \right]$$

where, *c*_*x*_ is cos(*x*) and *s*_*x*_ is sin(*x*). For a desired (*d*) target position and configuration of RFF in respect to {*O*} given by the vector:

3 $$^{\text{ATT}} \varvec{P}_{d} = \left[ {X_{d} Y_{d} Z_{d} \theta_{Xd} \theta_{Yd} \theta_{Zd} } \right]$$

where, $X_{d} Y_{d} Z_{d}$ are the Cartesian coordinates in respect to {O} and $\theta_{Xd} \theta_{Yd} \theta_{Zd}$ the Euler angles in respect to {*O*}, an analytical solution for the inverse kinematics of the ATT to provide the desired joint parameters is given by:

4 $$\begin{array}{*{20}c} {\theta_{1} = {\text{atan}}2\left( {X_{d} ,Y_{d} } \right)} \\ {\theta_{4} = \theta_{{Y_{d} }} } \\ {d_{2} = Z_{d} - \left( {l_{1} - l_{3} } \right)} \\ {d_{3} = \sqrt {X_{d}^{2} + Y_{d}^{2} } } \\ \end{array}$$

The RAFS system control architecture is reported in the Supplement S1.

RAFS navigation system consists of a virtual reduction (VR) software, optical tracking system (Polaris Spectra, NDI Inc., tracking accuracy 0.25 mm), and contact-less user controller (Leap Motion). The VR software displays the 3D models of the bone fragments created from the CT DICOM data. The surgeon moves the fragments using leap motion to virtually reduces the fracture.^6^,^9^ The optical tracking system provides real-time update of the 3D models through the optical tools attached to the orthopedic pins inserted into the bone fragments. Intra-operative imaging allows surgeon to monitor progress of the physical fracture reduction performed by the robotic system. The integration of image-guidance with the robotic system is introduced in the next section along with the new clinical workflow, and accurately described in Supplement S2.

RAFS system technical requirements have been defined based on the assessment results of earlier prototypes,^7^,^9^ and are summarized in Table 1.

**Table 1:** RAFS system technical data.

Parameter	Value
RFM positioning accuracy^11^	0.03 ± 0.01 mm (translational) 0.12 ± 0.01° (rotational)
CP positioning accuracy^7^	5 mm (translational) 5° (rotational)
ATT positioning accuracy	0.2 mm (translational) 0.3° (rotational)
RFM operational workspace^11^	±25 mm (*x*, *y* ,*z*), ± 17° (*ϑ*_*x*_, *ϑ*_*y*_, *ϑ*_*z*_)
CP operational workspace^7^	Cylindrical workspace ±350 mm (length), 300 mm (diameter)
RFM load capacity^7^	350 N (force), 12 Nm (torque)
ATT traction capacity	350 N (force)
Tracking system accuracy^11^	0.25 mm

*RFM* robotic fracture manipulator; *CP* carrier platform; *ATT* automated traction table

### RAFS Clinical Workflow

**Figure 4:**
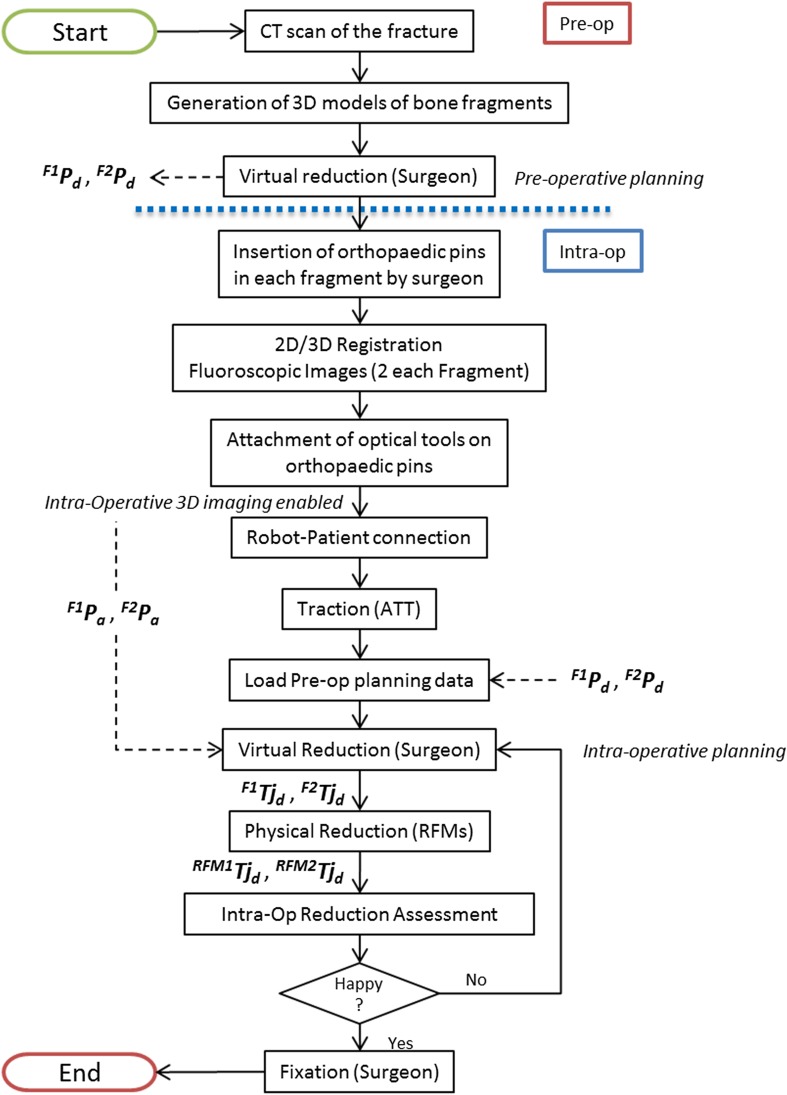
New clinical workflow for RAFS.

**Figure 5:**
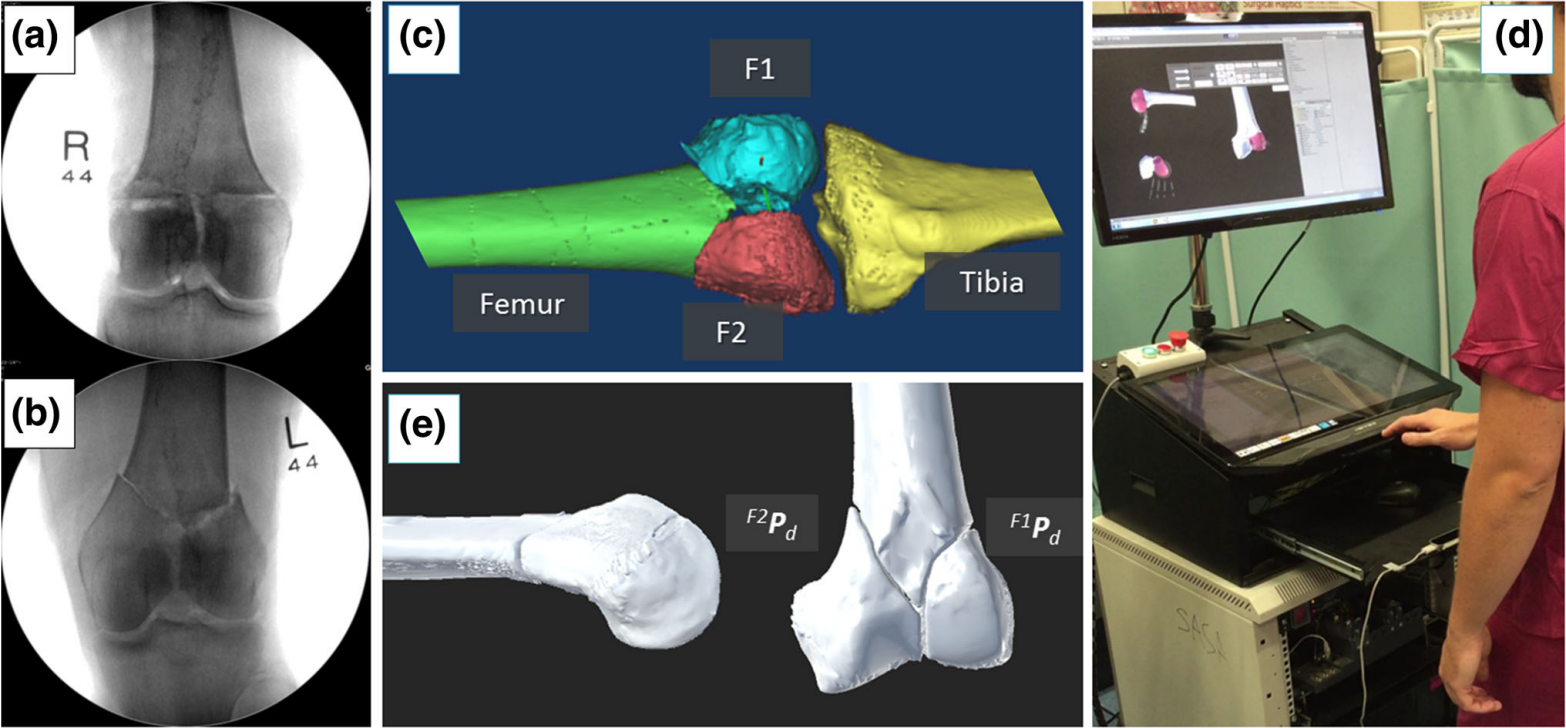
Pre-operative planning: CT-generated 3D models of a Y-shape 33-C1 fracture of a cadaveric specimen (a); a surgeon virtually reduces the fracture using the GUI (b); visual results of the virtual reduction, and generation of the pre-operative planning data ^*F*1^ *P*_*d*_ and ^*F*2^ *P*_*d*_ (c).

The new clinical workflow (Fig. 4) removes the issues of the earlier version, and allows full pre-operative planning. This involves a number of registrations and transformations which are described in the Supplement S2. Two different types of 2-fragment distal femur fractures have been considered in this study, i.e., articular Y-shape 33-C1 and T-shape 33-C1 fractures (Figs. 5a, 5b),^12^ as the large size of the fragments (medial and lateral condyles) can accommodate insertion of orthopedic manipulation pins through small incisions. The statistical validation of the robotic system is reported in Ref. 11.

A pre-operative CT scan of the fracture is taken, and the resulting dataset segmented to generate 3D models (CAD model) of each bone fragment (Fig. 5c). The models are imported in the reduction software and the surgeon virtually reduces the fracture using the GUI (Fig. 5d) by manipulating F1 (fragment 1) and F2 (fragment 2) to match FEM (femur—which remains fixed). This generates the desired final (*d*) poses for fragments F1 (^*F*1^ ***P***_*d*_) and F2 (^*F*2^ ***P***_*d*_) with respect to the femur (Fig. 5e). Pre-operative planning data are stored in the system and used for intra-operative robot motion calculations to achieve the physical reduction of the fracture.

**Figure 6:**
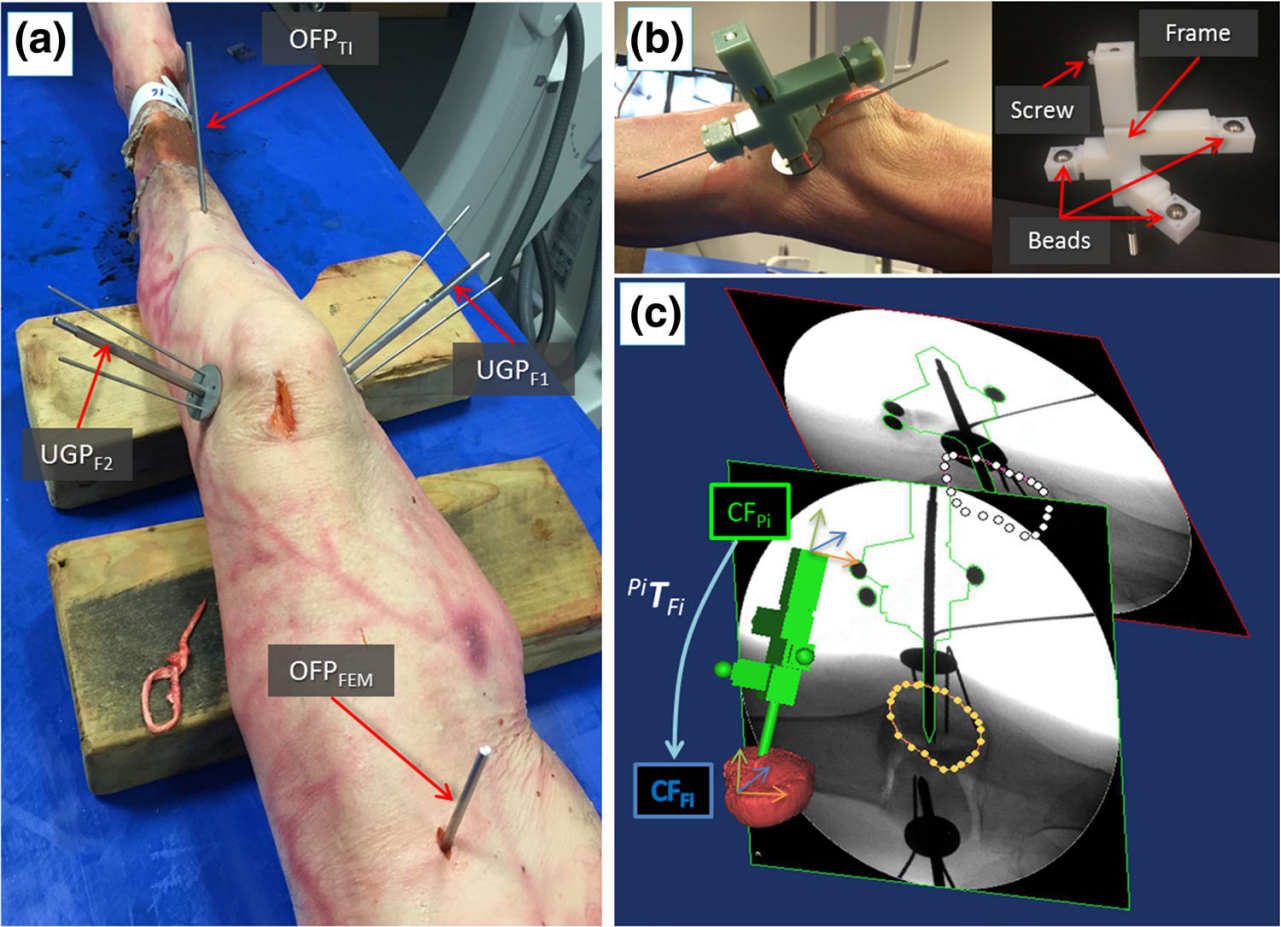
Intra-operative navigation. Cadaveric specimen with orthopedic pins inserted (a); example of a registration tool RT_UGP_ inserted into UGP pin (b); 2D/3D registration framework (c): 6DOF pose of the fluoroscopic images is estimated using the CAD model of the registration tool and the pin (green object); CT-generated model of the bone fragment (red object) is then registered with the fluoroscopic images; the relative pose between the coordinate frames of the fragment (CF_Fi_) and the inserted pin (CF_Pi_) is defined by the homogeneous transformation ^Pi^ *T*_Fi_.

In the operating theatre, 4 orthopedic pins are inserted into the bone fragments (Fig. 6a). Two UGPs are inserted into the fragments to be manipulated by the robot: i.e., UGP_F1_ is inserted in F1, UGP_F2_ in F2. UGPs are then firmly attached to the fragments using the AS. Two further pins (*D* = 4 mm, L = 135 mm) are inserted into the tibia (OFP_TI_) and the femur (OFP_FEM_). These pins have only two sections: the *threaded section* (M4 × 1, screwed into the bone) and the *tool section* (one-flat-face geometry), to which supporting registration and optical tools are fixed. We refer to these pins as one flat pins (OFPs). Pins are percutaneously inserted into the bones through small incisions, minimizing the soft tissue damage. The relative pose of the bone fragments is quite likely to change between the pre-operative CT scan is taken and the surgery starts. This is due to both the insertion of the pins into the bone fragments and small movements of the joint that might happen before the surgery. Therefore, the pre-operative pose of the fragments, i.e., the pose of the fragments at the time of the CT scan, can not be trusted during the surgical procedure. Pre-operative CT data are used only to generate the 3D models of the fragments and to allow the pre-operative planning of the procedure, and not to provide pose estimation of the fragments. Therefore, to enable intra-operative image guidance—i.e., the real-time updated pose of the bone fragments during the surgery—the relative position of each pin with respect to the bone fragment in which it is inserted needs to be calculated through intra-operative surgical registration. Once the relative pose of each pin-bone is known, and assuming that it does not change over the time (i.e., the object constituted by the pin and the bone fragment is considered rigid), the pose of each bone fragment can be updated in real-time by connecting an optical tool to the pin, as described below. This depicts the actual pose of each fragment (F1, F2, Tibia, and Femur) in the 3D space during the surgery.

**Figure 7:**
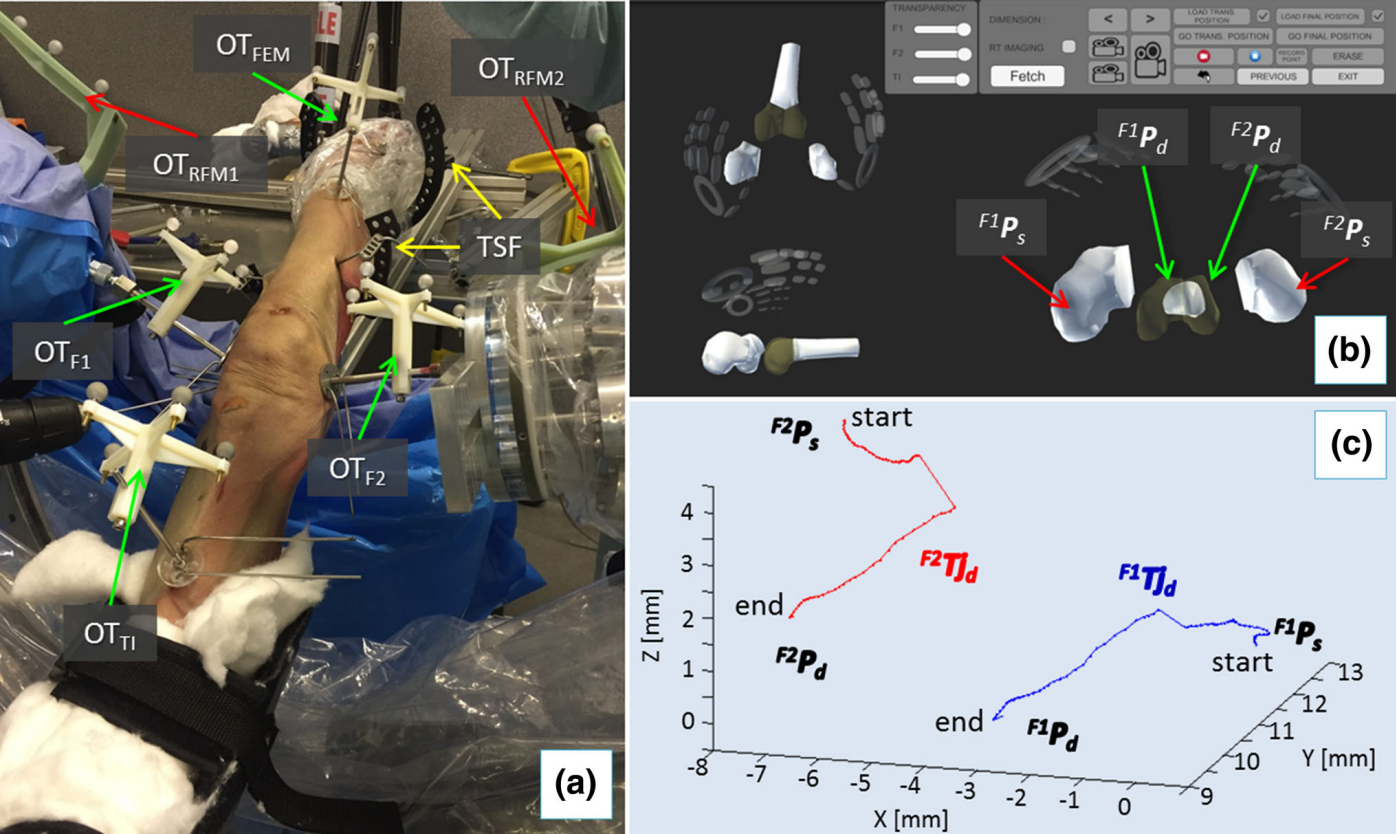
RAFS system in the cadaver laboratory. Optical tools attached to the orthopedic pins and RFMs allow intra-operative real-time imaging and closed-loop control of the system (a); pre-operative data are imported into reduction software and the surgeon proceeds with the intra-operative virtual reduction (b) generating the desired reduction trajectories ^*F*1^ *Tj*_*d*_ and ^*F*2^ *Tj*_*d*_ (c) for each fragment.

In this regard, custom-designed registration tools (RT_UGP_, RT_OFP_) have been prototyped. Each RT contains three stainless steel beads (radiopaque). RT_UGP_ has been designed to be rigidly connected in a unique way into UGPs, while RT_OFP_ fits only OFPs (Fig. 6b). RT_s_ have locking screws that ensure their perfect alignment with the pins. Two fluoroscopic images from different angles (i.e., 90°, 30°) of each fragment-pin-registration tool are taken (8 images in total). These images are imported into the reduction software, together with the CAD 3D models of the registration tools placed on the pins and the 3D models of the fracture generated by the pre-operative CT dataset. The proposed 2D/3D registration workflow is applied to each fragment-pin-registration tool and it involves (Fig. 7c):

(1) Estimation of a relative pose between the 2 fluoroscopic images using the registration tool: a bead-based registration algorithm^32^ is used to estimate the 6DOF pose of the fluoroscopic images with respect to the registration tool placed on the pin, using point correspondence between the known features in the CAD 3D model and in the images (Fig. 6c).

(2) Registration of the 3D model of the bone fragment to the fluoroscopic images: the contour of the bone fragment is segmented on the two fluoroscopic images, and a spline-based registration method^38^ is applied to minimize the distance between the projected contour of the 3D model of the bone fragment and the segmented 2D contour on each fluoroscopic image. After the registration, the relative pose between each pin and its fragment is known, and the homogeneous transformations ^Pi^ ***T***_Fi_ can be calculated (Fig. 6c and Supplement S2). ^Pi^ ***T***_Fi_ are considered to be constant during the operation (i.e., the relative pose of each pin-bone does not change over the time). The pose of each bone fragment is then updated in real-time by connecting an optical tool to its pin.

The femur is physically fixed to the operating table using two Taylor Spatial Frame (TSF) and k-wires, and registration tools are replaced by optical tools (Fig. 7a): optical tool OT_TI_ is placed on the pin (OFP_TI_) inserted in the tibia (TI), OT_FEM_ on the pin (OFP_FEM_) inserted in the femur (FEM), OT_F1_ on the pin (UGP_F1_) in fragment 1 (F1), and OT_F2_ on the pin (UGP_F2_) in fragment 2 (F2). Two optical tools (OT_RFM1_, OT_RFM2_) are also placed on the two RFMs. As the orthopedic pins were designed to be connected in a unique way to the optical tools (coincident coordinate frames, i.e., CF_Pi_ ≡ CF_OTi_), the optical tracker provides the actual (*a*) pose of each bone (^*F*1^ ***P***_*a*_, ^*F*2^ ***P***_*a*_, ^*T*I^ ***P***_*a*_, ^FEM^ ***P***_*a*_) by tracking the pin, thus enabling the intra-operative image guidance (see Supplement S2). The ATT is attached to the patient’s foot and the surgeon sets a desired pose ^ATT^ ***P***_*d*_ to apply traction to the knee joint. The surgeon monitors in real-time the actual pose of tibia and fragments F1 and F2 with respect to the femur using the navigation system. CP1 and CP2 position RFM1 and RFM2 close to the orthopedic pins UGP_F1_ and UGP_F2_, (poses described by OT_F1_ and OT_F2,_ respectively). RFM1 is now connected to UGP_F1_, RFM2 to UGP_F2_, and F1 and F2 are in their initial starting (*s*) poses (^*F*1^ ***P***_***s***_, ^*F*2^ ***P***_***s***_). Results of the pre-operative planning ^*F*1^ ***P***_*d*_ and ^*F*2^ ***P***_*d*_ are uploaded into the intra-operative procedure. The surgeon proceeds with the intra-operative virtual reduction of the fracture (Fig. 7b), generating the desired trajectories ^*F*1^ ***Tj***_*d*_ and ^*F*2^ ***Tj***_*d*_ to take F1 and F2 from their initial starting poses (^*F*1^ ***P***_***s***_, ^*F*2^ ***P***_***s***_) to the final ones (^*F*1^ ***P***_*d*_, ^*F*2^ ***P***_*d*_) (Fig. 7c). The corresponding desired trajectories in the task space for the RFMs ^*RFM*1^ ***Tj***_*d*_ and ^*RFM*1^ ***Tj***_*d*_ are calculated (see Supplement S2) to achieve the fracture reduction. The RFMs execute the desired movements for F1 and F2 while FEM remains static. The optical imaging system is responsible for 3D visualizing of the fracture fragments in real-time replacing the use of intra-operative fluoroscopy. When the reduction is acceptable, the surgeon fixates the fracture.

### Experimental Evaluation

RAFS surgical system has been evaluated on 9 human cadaveric specimens that include 3 of female and 6 of male subjects (approved by the National Research Ethics Committee, REC Reference: 15/WM/0038, UK) with distal femur fractures (see Supplement Video). Fractures were imaged with a SOMATOM Sensation 16 (Siemens Healthcare) CT scanner with a voxel size of 0.58 mm × 0.58 mm × 0.75 mm. Intra-operative fluoroscopic images were taken with an OEC Fluorostar (GE) C-arm. Each fluoroscopic image has been calibrated to correct the spatial image distortion,^8^,^30^ and improve the image processing accuracy. The metrics chosen for the RAFS system evaluation were (1) the fracture reduction accuracy expressed as the root-mean-squared-error (RMSE) measured after the physical reductions; (2) the force/torque involved in the physical reductions, i.e., the traction force applied by the ATT and the force/torque applied by the RFM_S_; (3) the UGP-RFM connection stability measured as the relative pose displacement between the UGP and the RFM end-effector; and (4) the surgical procedure time, i.e., the intra-operative time including pins insertion, 2D/3D registration, robot setup, intra-operative virtual reduction, and robotic fracture reduction.

Nine fresh frozen cadaver specimens (including hip and foot)—namely 4 right and 5 left lower limbs from both male (*N* = 6) and female (*N* = 3) with average age of 87 ± 11 years - were used for the study. An orthopedic surgeon fractured the distal part of each femur creating Y-shape 33-C1 fractures in 5 specimens and T-shape 33-C1 in 4 specimens. The proposed clinical workflow was applied to each specimen to reduce the fracture. The average registration error resulted to be 1.15 ± 0.8 mm.^10^

After each physical reduction, the surgeon checked the result intra-operatively using the navigation system and temporarily fixated the fracture using k-wires, ensuring the immobilization of the fragments during the assessment of the reduction (see Supplement Video). Assessment of closed fracture reductions in clinical practice is undertaken by the operating surgeon with the aid of fluoroscopic images.^12^ Following the temporary fixation, three fluoroscopic images of the reduced fracture were taken: one in the coronal plane (anteroposterior), one in the sagittal-lateral plane, and one in the sagittal-medial plane. The assessment of reduction accuracy was completed using the fluoroscopic images and the Sante DICOM Viewer (Santesoft). A displaced fragment causes a deviation from the normal alignment which can be quantified by measurements of translation and angulation on the fluoroscopic images in both coronal and sagittal planes.^3^ Translational accuracy ∆*T*_*i*_, defined as separation of two points, was measured by a clinician at several points where displacement between one manipulated fragment (*T*_Fi_) and the femur (*T*_*Ri*_ reference) was perceived to be the greatest. For each fragment, 12 data points were taken from two different fluoroscopic images (six points in the coronal plane and six in the lateral plane) to determine the average translational error. Rotational accuracy was measured as the difference between the axis defined by the femur *ϑ*_*Ri*_ (reference) and the axis defined by the fragment *ϑ*_Fi_. For each fragment 2 data points were taken from two different fluoroscopic images (one in the coronal plane, one in the lateral plane) to determine the average rotational error.

## Results

**Figure 8:**
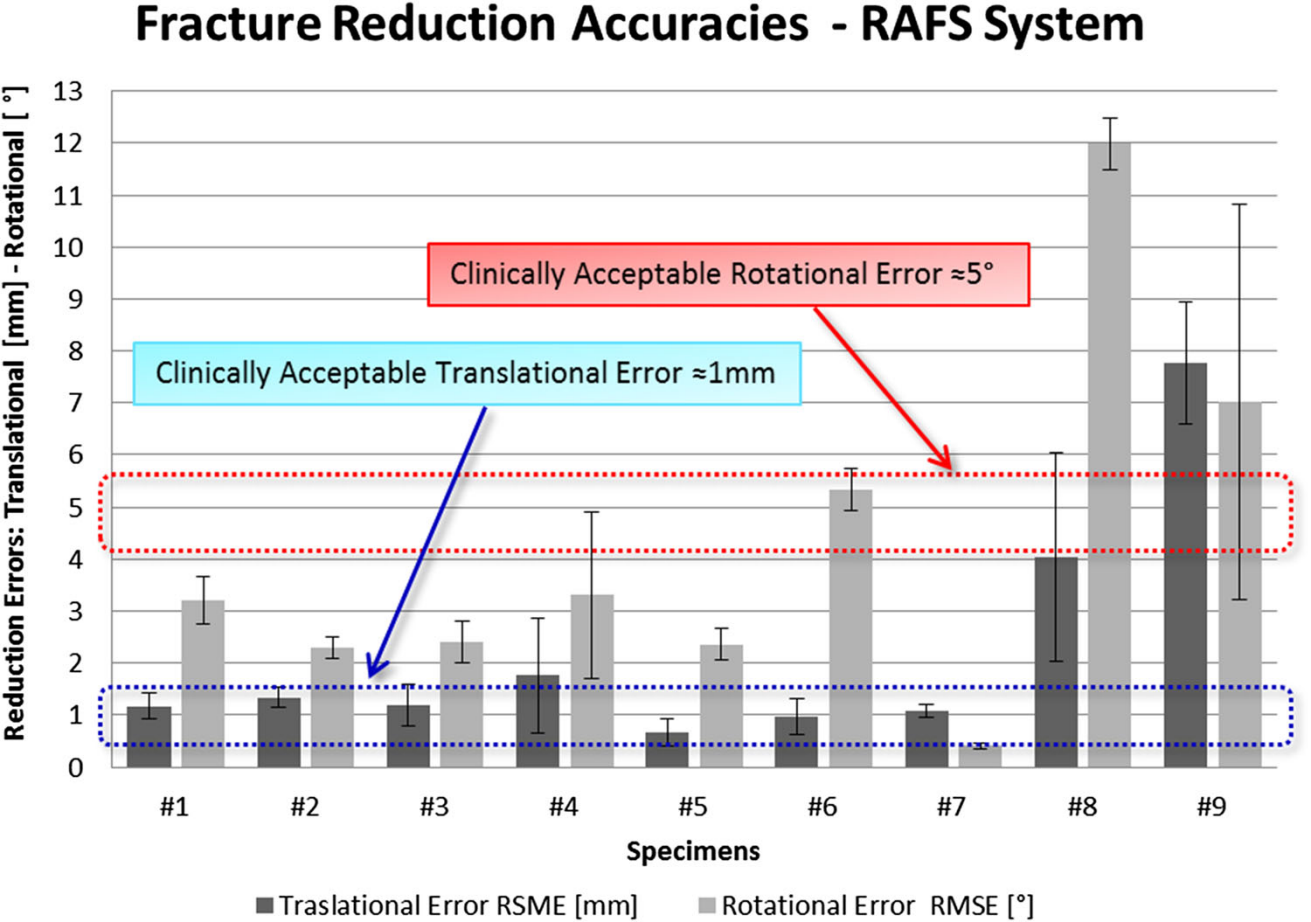
Fracture reduction accuracies achieved using the RAFS system on nine cadaveric specimens. The RAFS system was able to reduce distal femur fractures with acceptable clinical accuracy (Translational: ≈1 mm, blue rectangle—Rotational: ≈5°, red rectangle) in specimens #1, #2, #3, #5, and #7. Borderline—still acceptable—reduction accuracy was measured in specimens #4 and #7. The RAFS system was unsuccessful in reducing the fractures in specimens #8 and #9.

Experimental results are summarized in Table 2. The RAFS system showed clinically acceptable reduction values (≈1 mm, ≈5°) on both Y- and T-shape 33-C1 fractures in 5 specimens, namely #1, #2, #3, #5, and #7 as shown in Fig. 8. Columns “F1 RMSE” and “F2 RMSE” reports reduction accuracies for fragment 1 and fragment 2 respectively (in terms of root-mean-squared error) with regard to each specimen, showing that the system can accomplish a good reduction accuracy avoiding large deviations from the desired reduction. Figure 8 plots the average reduction accuracies of F1 and F2 for each specimen, providing a visual representation of the overall reduction accuracies achieved using the RAFS system on each specimen, compared with the clinically acceptable values. This is also summarized in the “Overall” column of Table 2.

**Table 2:** Cadaveric trials results.

Specimen	Reduction accuracy			RFMs-pins displacement		Traction force^d^	Manipulation force/torque^e^	Surgery time
	F1 RMSE^a^	F2 RMSE^a^	Overall^b^	RFM1 UGP_F1_^e^	RFM2 UGP_F2_^e^			
#1—T,R	1.41 ± 0.30 mm 3.12 ± 0.40°	0.93 ± 0.20 mm 3.30 ± 0.50°	A	2.70 mm 3.10°	2.65 mm 4.40°	10.8 ± 2.3 N	69.9 ± 4.4 N 4.8 ± 0.4 Nm	119 min
#2—Y,R	1.83 ± 0.10 mm 2.40 ± 0.30°	0.85 ± 0.30 mm 2.20 ± 0.10°	A	1.37 mm 1.60°	3.30 mm 2.70°	51.4 ± 2.8 N	113.1 ± 5.4 N 3.2 ± 0.3 Nm	131 min
#3—T,L	1.00 ± 0.40 mm 2.40 ± 0.20°	1.38 ± 0.40 mm 2.40 ± 0.60°	A	2.10 mm 1.80°	2.80 mm 3.45°	24.0 ± 0.8 N	18.0 ± 0.5 N 1.60 ± 0.1 Nm	132 min
#4—Y,L	0.69 ± 0.26 mm 1.75 ± 0.10°	2.83 ± 1.94 mm 4.88 ± 3.10°	B	2.3 mm 3.35°	3.68 mm 23.0°	12.5 ± 3.1 N	94.6 ± 5.1 N 6.83 ± 0.6 Nm	119 min
#5—T,L	0.51 ± 0.12 mm 2.72 ± 0.01°	0.82 ± 0.39 mm 2.01 ± 0.58°	A	2.63 mm 1.07°	5.99 mm 5.52°	51.6 ± 24 N	147 ± 10 N 6.31 ± 0.2 Nm	117 min
#6—T,R	0.79 ± 0.11 mm 3.43 ± 0.22°	1.15 ± 0.60 mm 7.24 ± 0.56°	B	2.20 mm 3.07°	2.97 mm 1.34°	10.4 ± 1.2 N	82.7 ± 7.5 N 1.96 ± 0.3 Nm	127 min
#7—Y,L	1.04 ± 0.25 mm 0.12 ± 0.05°	1.13 ± 0.01 mm 0.69 ± 0.04°	A	2.79 mm 2.29°	2.81 mm 2.46°	45.6 ± 5.1 N	25.9 ± 7.4 N 3.24 ± 0.6 Nm	123 min
#8—Y,L	7.13 ± 3.63 mm 20.7 ± 0.81°	0.95 ± 0.37 mm 3.28 ± 0.18°	N	2.84 mm 18.1°	0.89 mm 0.91°	8.3 ± 2.6 N	55.9 ± 11.9 N 1.91 ± 0.4 Nm	119 min
#9—Y,R	3.44 ± 0.82 mm 1.14 ± 0.16°	12.1 ± 1.54 mm 12.9 ± 7.42°	N	1.85 mm 2.48°	2.73 mm 8.85°	11.6 ± 5.1 N	74.5 ± 8.1 N 2.12 ± 0.5 Nm	107 min

^a^Reduction accuracy is described by the translational root-mean-squared-error (RMSE in mm) and the rotational root-mean-squared-error (RMSE in degrees)

^b^Qualitative evaluation of the reduction accuracy considering both F1 and F2. The overall reduction accuracy is considered acceptable (A) for reduction values of F1 and F2 ≈1 mm and ≈5°. Slightly higher reduction values bring to a borderline (B) reduction, although still clinically acceptable. Higher reduction values of F1 and F2 are considered clinically not acceptable (N)

^c^Connection stability of RFMs and UGPs is described by the maximum translational (in mm) and rotational (in degrees) displacement between the UGP_F1_ connected to RFM1 end-effector and the UGP_F2_ connected to RFM2 end-effector

^d^Average traction (measured in N) applied by the automated traction table (ATT) during the surgical procedure

^e^Resultant average forces (N) and torques (Nm) applied by the Robotic Fracture Manipulators (RFM_S_) during the surgical procedure

*T* T-shape 33-C1 fracture; *Y* Y-shape 33-C1 fracture; *R* right limb; *L* left limb

Specimens #4 and #6 presented average residual error (RMSE) of 1.76 ± 1.1 mm/3.32 ± 1.6° (specimen #4) and 0.97 ± 0.35 mm/5.33 ± 0.4° (specimen #6) (Fig. 8), resulting in sub-optimal—although still acceptable—reduction. A higher residual error for F2 (2.83 ± 1.94 mm/4.88 ± 3.10°) in specimen #4 is due to a failure in the gripping system (GS) as shown by the relative rotation between UGP_F2_ and RFM2 of 23° (UGP_F2_ rotated inside GS) reported in column “RFM2 UGP_F2_”of Table 2. The failure occurred because of a higher than the average torque applied by RFM2 during the manipulation of F2 (6.83 ± 0.6 Nm, see column “Manipulation Force/Torque” of Table 2).

A residual rotational error of 7.24 ± 0.56° was measured for F2 in specimen #6. The initial dislocation of this fragment was high (rotation over 45°) and the RFM2 was not able to achieve a sufficient reduction as the required movement of the RFM2 was beyond the designed workspace capabilities.

Reduction accuracy for specimens #8 and #9 can’t be considered acceptable. Considering specimen #8, the reduction accuracy achieved for fragment 1 was 7.13 ± 3.63 mm (translational) and 20.7 ± 0.81° (rotational). In specimen #9, the reduction accuracy for fragment 2 was 12.1 ± 1.54 mm (translational) 12.9 ± 7.42° (rotational), much higher than the acceptable values of 1 mm and 5°. Again, the malreductions obtained for fragment 1 of specimen #8 and fragment 2 of specimen #9, are related with the failure of the GS, not being able to keep the UGPs stationary inside the RFMs. The relative displacement between UGP and RFM (see column “RFMs-Pins Displacement” in Table 2), due to soft tissue-related forces and torques, was measured during each reduction comparing the relative pose of each UGP (provided by OT_F1_ and OT_F2_) and the connected RFM (provided by OT_RFM1_ and OT_RFM2_). Whilst rotational displacements were used as a metric to evaluate safety of the connection between UGP and GS (i.e., RFM), linear displacements describe the bending of the UGP at the *gripping* section. Average linear and rotational displacements of 2.7 mm (maximum 5.99 mm) and rotational 4.9° (maximum 23° measured in specimen#4), respectively, were measured.

The ATT supported the fragment manipulation and facilitated the reduction of the fractures. The average traction force applied by the ATT measured during the nine reductions was 25.1 ± 17.8 N (maximum 51.6 N). Specimen-specific traction values are reported in column “Traction Force”, while manipulation forces and torques exerted by the RFMs during the reduction procedure are shown in column “Manipulation Forces/Torques” of Table 2.

Finally, the surgical times to reduce the fracture on each specimen using the RAFS system are reported in column “Surgery Time” of Table 2. The average surgical time to reduce a DFF with the RAFS system was 123 ± 7 min, slightly higher than the open-procedure (≈100 min).^15^ Only intra-operative time was considered in this study as it directly affects the patient (e.g., duration of the anesthesia) and the operating theatre management (e.g., operating theatre occupancy rate). Pins insertion took 33 ± 3 min; 2D/3D registration 52 ± 6 min; robot setup 27 ± 3 min; intra-operative virtual reduction 2 ± 1 min; and physical reduction 9 ± 3 min.

## Discussion

Cadaveric trials demonstrated the potential that the RAFS system can have for joint fracture surgery. Image-guidance and robotic assistance are combined to improve the surgical management of distal femur fractures. CT images (3D models of the fracture) are used both to guide the surgeon in pre-planning the surgical procedure (virtual reduction), and to generate the motion commands for the robotic system. Robotic assistance overcomes the high forces involved in the manipulation of bone fragments to achieve the physical reduction of the fracture with high accuracy, which would not be possible otherwise (i.e., through percutaneous manual reduction).

The metric chosen for the reduction accuracy evaluation, i.e., RMSE, gives an account of how far the manipulated fragments are from the desired, reduced, position. Results (Table 2; Fig. 8) showed that clinically acceptable reduction was achieved in 5 specimens (#1 to #7). Two specimens (#4, #6) resulted in sub-optimal reduction, although still clinically acceptable. The GS failed during the reduction of specimen #4 due to the high torque applied by RFM2 during the manipulation of F2 (6.83 ± 0.6 Nm). High forces/torques values depend on the soft tissue opposing the reduction, which can cause the UGPs to bend and—as in this case—the gripping system to fail, increasing the reduction error. The force/torque applied by the RFMs during the cadaveric trials was on average 75.7 N/3.5 Nm, with maximum values of 147 N/6.83 Nm (Table 2, column “Manipulation Force/Torque”). Forces measured during cadaveric trials are almost up to nine times higher than the force data collected with the earlier prototypes in laboratory trials on phantoms (16.5 N), and torque data almost 5 times higher (1.5 Nm).^7^,^9^ This also explains the slightly lower reduction accuracy—on average—of the new RAFS system with respect to the earlier prototypes (1.15 mm/1.3°).^7^,^9^

The control system was able to compensate the displacement between the RFMs and UGPs (Table 2, column “RFMs-Pins Displacement”) by re-calculating the homogenous transformations ^RFM1^ ***T***_UGPF1_ and ^RFM2^ ***T***_UGPF2_ at each processing time step (see Supplement S2), achieving clinically acceptable reduction accuracy in specimens #1 to #7. The system was not able to reduce the fractures in specimens #8 and #9 for two reasons: (1) the initial dislocation of the fragments was high (rotation of up to 45°); and (2) the gripper system failed. The conjunction of the two has resulted in movement of the RFMs beyond the designed workspace capabilities. Operational workspace of RFMs and the gripper system design need further attention in future developments of the RAFS system. The linear displacements of 2.7 mm (average) and 5.99 mm (maximum, specimen #5) measured between the RFMs and UGPs during cadaveric trials (Table 2, column “RFMs-Pins displacements”) were obtained for applied forces/torques (Table 2, column “Manipulation Force/Torque”) of 76 N/3.5 N (average) and 147 N/6.31 Nm (maximum, specimen #5). Similar to the *gripping* section, the other UGP’s sections bend when a high load is applied. The control system cannot compensate this displacement as it can’t be estimated during the surgical procedure (a further optical tool on the *threaded* section would be needed). This, of course, affects the physical reduction accuracy of the fracture. A mathematical model of the UGP’s force–displacement relation can be created based on FEA simulations. In the future, this model can be included in the RAFS control system allowing a real-time estimation and compensation of the UGP displacement (including the *gripping* section) based on force/torque feedback provided by the load cells mounted on the RFMs.

Comparative evaluation of forces applied by ATT (Table 2, column “Traction Force”) and RFMs (Table 2, column “Manipulation Force/Torque”) showed that the minimum manipulation force applied by RFMs to reduce the fracture was measured with a traction force (ATT) of about 35 ± 10 N (e.g., specimens #3, #7). Lower traction forces have resulted in a more difficult fragment manipulation. The joint was still compressed, and the fragments stuck between tibia and femur required higher manipulation forces (specimen #1, #4, #6, #8, #9). Higher traction forces resulted in an over-tension of the knee ligaments (i.e., ACL, PCL, MCL, LCL),^37^ increasing the stiffness of the joint and the force required to manipulate the fragments (specimen #2, #5). No correlation between the traction forces and the manipulation torque was identified. However, a qualitative correlation between manipulation torques and the knee flexion angle, i.e., the angle between femur and tibia on the lateral plane (provided by the optical tools OT_TI_ and OT_FEM_) was found. Flexion of the knee at about 20° resulted in a lower required torque applied by the RFMs (specimen #3, #8). Data collected during the fracture reduction of specimen #3, perfectly summarizes the findings above: traction force of 24.0 ± 0.8 N and knee flexion of 20° resulted in the lowest manipulation force/torque measured during the whole cadaveric study. This confirms the clinical practice and the use of carbon triangles to support the leg and relax the knee joint muscles during the procedure.^12^

The surgical registration is a key part of the clinical workflow. It was successfully executed on all the 9 specimens, showing that it is reliable and effective, and can achieve intra-operative registration with high level of accuracy, providing a registration error of only 1.15 ± 0.8 mm. Comparison between the registration accuracy and the reduction accuracy data in specimens #1, #2, #3, #5, and #7 (i.e., clinically acceptable reduction is achieved, no GS failure, no operational workspace issues) shows that the registration considerably affects the reduction accuracy, introducing the high majority of the final reduction error. This is mainly due to the manual user interaction required to perform the registration, which not only negatively affects the registration accuracy, but also slows down the whole procedure. The surgical registration is currently overly time consuming (≈50 min) at this stage of development, as user interaction is required to identify the location of anatomical landmarks and surfaces to carry out the registration. The manual interaction can be minimized by automating the surgical registration, resulting in both reduced registration time (ideally performed in few seconds), and increased registration and reduction accuracies. The identification of the anatomical landmarks can be automated using pattern matching algorithms looking for the desired features.^16^ At this stage, the segmentation of the bone fragments in the lateral fluoroscopic image requires an extensive manual intervention. One of the main issues encountered was the overlap of the bone fragments in the fluoroscopic image taken from the lateral view. An optimized positioning of the fluoroscope (e.g., in Ref. 34) in order to obtain images with minimum overlap of the fragments might enable an automatic segmentation of the bones in the fluoroscopic images. Also, the registration algorithms can be implemented on a graphic processing unit (GPU) as proposed in, Ref. 26 speeding up the processing time. The whole surgical procedure would benefit from this, as the surgery time could be potentially reduced to about 1 h—much below the standard open-reduction procedure (≈100 min).

At the current stage of development, the RAFS system is applicable to fractures in those who have reached skeletal maturity—which is above the age of 17–18 in both sexes^19^—following the clinical workflow described in this manuscript. Below this age, surgical management of skeletal trauma is different to that of adult trauma due to a combination of reasons including the higher rate of bone healing, differences in biomechanical properties of the bone, and considerations for the growth (epiphyseal) plates.^24^ Overall there is a higher threshold for operative intervention as many fractures, even displaced ones, remodel well with plaster treatment despite malreduction.^4^ For those that do require operative intervention, the indications are usually that of neurovascular compromise,^5^ or gross intra-articular incongruity in a characteristic type of joint fractures (e.g., distal tibial).^22^ In both cases reduction is usually achieved closed and fixation carried out percutaneously. In such cases it may be theorized that the RAFS system may be a suitable tool to aid reduction. However certain technical obstacles, such as the size of the fragments, and pin fixation in the softer pediatric bone would need to be overcome first.

In summary, cadaveric trials demonstrated the accuracy and effectiveness of the RAFS system, and its applicability and usability in clinical environment, thus paving a way towards minimally invasive fracture surgeries. Cadaveric trials provided valuable data for future improvements. Methods to further improve the success ratio will include a redesign of the RFMs which can be made more compact but with a larger workspace. The gripper system will be resigned to provide a more stable gripping to avoid displacements between pins and RFMs that can cause sub-optimal reductions. Future work will be focused on the automation of the intra-operative surgical registration to make the whole surgical procedure quicker and more accurate. On the control side, a hybrid force-position control and mathematical model of the UGP could enable automatic adjustment of the fragment positioning based on force-torque feedback .

## Electronic supplementary material

Below is the link to the electronic supplementary material.

Supplementary material 1 (MP4 183330 kb)

Supplementary material 2 (PNG 269 kb)

Supplementary material 3 (PNG 35 kb)

Supplementary material 4 (PNG 211 kb)

Supplementary material 5 (PNG 451 kb)

## References

[1] Botchu R, Ravikumar K (2008). Radiation exposure from fluoroscopy during fixation of hip fracture and fracture of ankle: effect of surgical experience. Indian J. Orthop..

[2] Buschbaum J, Fremd R, Pohlemann T, Kristen A (2014). Computer-assisted fracture reduction: a new approach for repositioning femoral fractures and planning reduction paths. Int. J. Comput. Assist. Radiol. Surg..

[3] Cole RJ, Bindra RR, Evanoff BA, Gilula LA, Yamaguchi K, Gelberman RH (1997). Radiographic evaluation of osseous displacement following intra-articular fractures of the distal radius: reliability of plain radiography versus computed tomography. J. Hand Surg..

[4] Crawford SN, Lee LSK, Izuka BH (2012). Closed treatment of overriding distal radial fractures without reduction in children. J. Bone Joint Surg. Am..

[5] Dabis J, Daly K, Gelfer Y (2016). Supracondylar fractures of the humerus in children: review of management and controversies. Orthop. Muscul. Syst. Curr. Res..

[6] Dagnino, G., I. Georgilas, F. Girault, P. Tarassoli, R. Atkins, and S. Dogramadzi. Surgical pre-planning for robot-assisted fracture surgery. In: The Hamlyn Symposium, 2016.

[7] Dagnino, G., I. Georgilas, P. Köhler, R. Atkins, and S. Dogramadzi. Image-based robotic system for enhanced minimally invasive intra-articular fracture surgeries. In: Robotics and Automation (ICRA), 2016 IEEE International Conference on (pp. 696–701). IEEE, 2016. doi:10.1109/ICRA.2016.7487196.

[8] Dagnino, G., L. S. Mattos, and D. G. Caldwell. New Software Tools for Enhanced Precision in Robot-Assisted Laser Phonomicrosurgery. In: Engineering in Medicine and Biology Society (EMBC), 2012 Annual International Conference of the IEEE (pp. 2804–2807). IEEE. 2012. doi:10.1109/EMBC.2012.6346547. 10.1109/EMBC.2012.6346547 23366508

[9] Dagnino G, Georgilas I, Köhler P, Morad S, Atkins R, Dogramadzi S (2016). Navigation system for robot-assisted intra-articular lower-limb fracture surgery. Int. J. Comput. Assist. Radiol. Surg..

[10] Dagnino G, Georgilas I, Morad S, Gibbons P, Tarassoli P, Atkins R, Dogramadzi S (2017). Intra-operative fiducial-based CT/fluoroscope image registration framework for image-guided robot-assisted joint fracture surgery. Int. J. Comput. Assist. Radiol. Surg..

[11] Dagnino G, Georgilas I, Tarassoli P, Atkins R, Dogramadzi S (2015). Vision-based real-time position control of a semi-automated system for robot-assisted joint fracture surgery. Int. J. Comput. Assist. Radiol. Surg..

[12] Distal Femur Fracture—Reduction and Fixation. https://www2.aofoundation.org/.

[13] Dobbe JGG, Strackee SD, Schreurs AW, Jonges R, Carelsen B, Vroemen JC, Grimbergen CA, Streekstra GJ (2011). Computer-assisted planning and navigation for corrective distal radius osteotomy, based on pre- and intraoperative imaging. IEEE Trans. Biomed. Eng..

[14] Gaston P, Will EM, Keating JF (2005). Recovery of knee function following fracture of the tibial plateau. J Bone Joint Surg Br.

[15] Gavaskar AS, Tummala NC, Krishnamurthy M (2011). Operative management of Hoffa fractures–a prospective review of 18 patients. Injury.

[16] George AK, Sonmez M, Lederman RJ, Faranesh AZ (2011). Robust automatic rigid registration of MRI and X-ray using external fiducial markers for XFM-guided interventional procedures. Med. Phys..

[17] Graham, A. E., S. Q. Xie, K. C. Aw, W. L. Xu, and S. Mukherjee. Design of a Parallel Long Bone Fracture Reduction Robot with Planning Treatment Tool. In Intelligent Robots and Systems, 2006 IEEE/RSJ International Conference on (pp. 1255–1260). IEEE, 2006. doi:10.1109/IROS.2006.281885.

[18] Graham AE, Xie SQ, Aw KC, Mukherjee S, Xu WL (2008). Bone-muscle interaction of the fractured femur. J. Orthop. Res. Off. Publ. Orthop. Res. Soc..

[19] Javangula P, Uloopi K, Vinay C, Rayala C, Kumar N, Chandra S (2017). Comparison of middle phalanx of the middle finger and cervical vertebrae as skeletal maturity indicators. Indian J Dent Sci.

[20] Joskowicz L, Milgrom C, Simkin A, Tockus L, Yaniv Z (1998). FRACAS: a system for computer-aided image-guided long bone fracture surgery. Comput. Aided Surg..

[21] Marsh J.L. Rockwood And Green’s Fractures In: Adults. Wolters Kluwer, 2015.

[22] Masquijo JJ, Allende V (2011). Triplane fracture of the distal femur: a case report. J. Pediatr. Orthop..

[23] Mathew G, Hanson BP (2009). Global burden of trauma: need for effective fracture therapies. Indian J. Orthop..

[24] Naranje SM, Stewart MG, Kelly DM, Jones TL, Spence DD, Warner WC, Beaty JH, Sawyer JR (2016). Changes in the treatment of pediatric femoral fractures: 15-year trends From United States Kids’ Inpatient Database (KID) 1997–2012. J. Pediatr. Orthop..

[25] NHS Digital. Hospital Episode Statisticsat http://content.digital.nhs.uk/hes.

[26] Otake Y, Armand M, Armiger RS, Kutzer MD, Basafa E, Kazanzides P, Taylor RH (2012). Intraoperative image-based multiview 2D/3D registration for image-guided orthopaedic surgery: incorporation of fiducial-based C-arm tracking and GPU-acceleration. IEEE Trans. Med. Imaging.

[27] Raabe D, Dogramadzi S, and Atkins R. Semi-automatic percutaneous reduction of intra-articular joint fractures—an initial analysis. In: Robotics and Automation (ICRA), 2012 IEEE International Conference on. IEEE, 2012. doi:10.1109/ICRA.2012.6225076.

[28] Rammelt S, Amlang M, Barthel S, Gavlik J-M, Zwipp H (2010). Percutaneous treatment of less severe intraarticular calcaneal fractures. Clin. Orthop..

[29] Rüedi T, Buckley R, Morgan C (2007). AO principles of fracture management, books and DVD.

[30] Schumann, S., X. Dong, M. Puls, L. P. Nolte, and G. Zheng. Calibration of C-arm for orthopedic interventions via statistical model-based distortion correction and robust phantom detection. In: Biomedical Imaging (ISBI), 2012 9th IEEE International Symposium on. IEEE, 2012. doi:10.1109/ISBI.2012.6235777.

[31] Tang P, Hu L, Du H, Gong M, Zhang L (2012). Novel 3D hexapod computer-assisted orthopaedic surgery system for closed diaphyseal fracture reduction. Int J Med Robot.

[32] Tsanaka, A., I. Georgilas, G. Dagnino, and S. Dogramadzi. Intra-operative x-ray dimensional calibration using orthopaedic implants. In: 5th IEEE RAS & EMBS International Conference on Biomedical Robotics and Biomechatronics, 14–15(1), 2014.

[33] Wang J, Han W, Lin H (2013). Femoral fracture reduction with a parallel manipulator robot on a traction table. Int. J. Med. Robot. Comput. Assist. Surg..

[34] Wang X, Yang J, Chen Y, Ai D, Hu Y, Wang Y (2014). Optimal viewing angle determination for multiple vessel segments in coronary angiographic image. IEEE Trans. Nucl. Sci..

[35] Warisawa S, Ishizuka T, Mitsuishi M, and Sugano N. Development of a femur fracture reduction robot. In: Robotics and Automation, 2004. Proceedings. ICRA’04. 2004 IEEE International Conference on. Vol. 4. IEEE, 2004. doi:10.1109/ROBOT.2004.1308896.

[36] Westphal R, Winkelbach S, Wahl F, Gösling T, Oszwald M, Hüfner T, Krettek C (2009). Robot-assisted long bone fracture reduction. Int. J. Robot. Res..

[37] Woo SL, Debski RE, Withrow JD, Janaushek MA (1999). Biomechanics of knee ligaments. Am. J. Sports Med..

[38] Zhang X, Zheng G, Langlotz F, Nolte L-P (2006). Assessment of spline-based 2D-3D registration for image-guided spine surgery. Minim. Invasive Ther. Allied Technol..

