# Supplementary figures and images for: Image-Guided Surgical Robotic System for Percutaneous Reduction of Joint Fractures
Source: Ann Biomed Eng. 2017 Aug 16;45(11):2648–62. doi: 10.1007/s10439-017-1901-x (PMC5663813; doi:10.1007/s10439-017-1901-x)

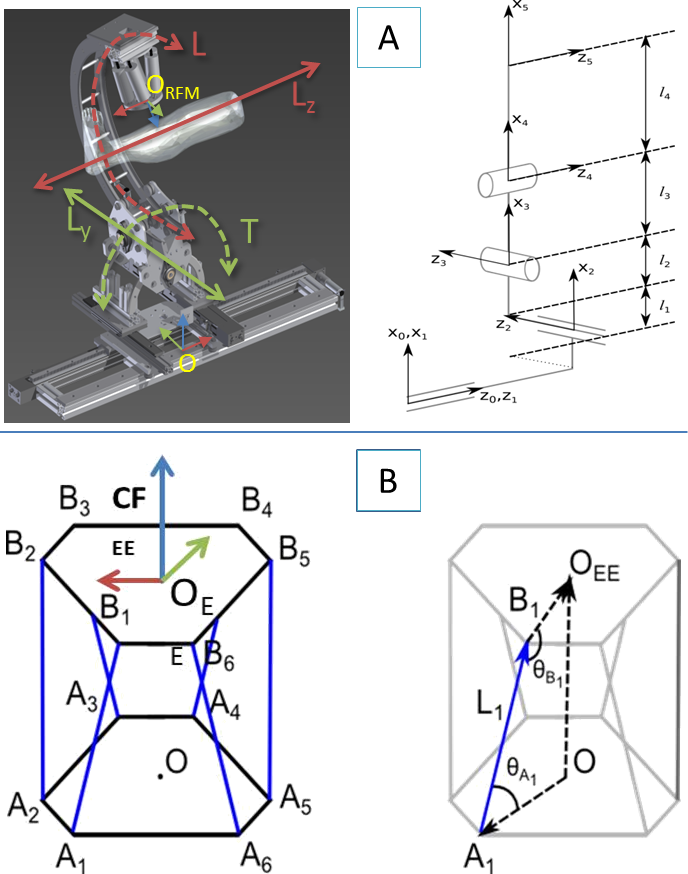

Supplement: Supplementary file 2 — Supplementary material 2 (PNG 269 kb) [file 10439_2017_1901_MOESM2_ESM.png]

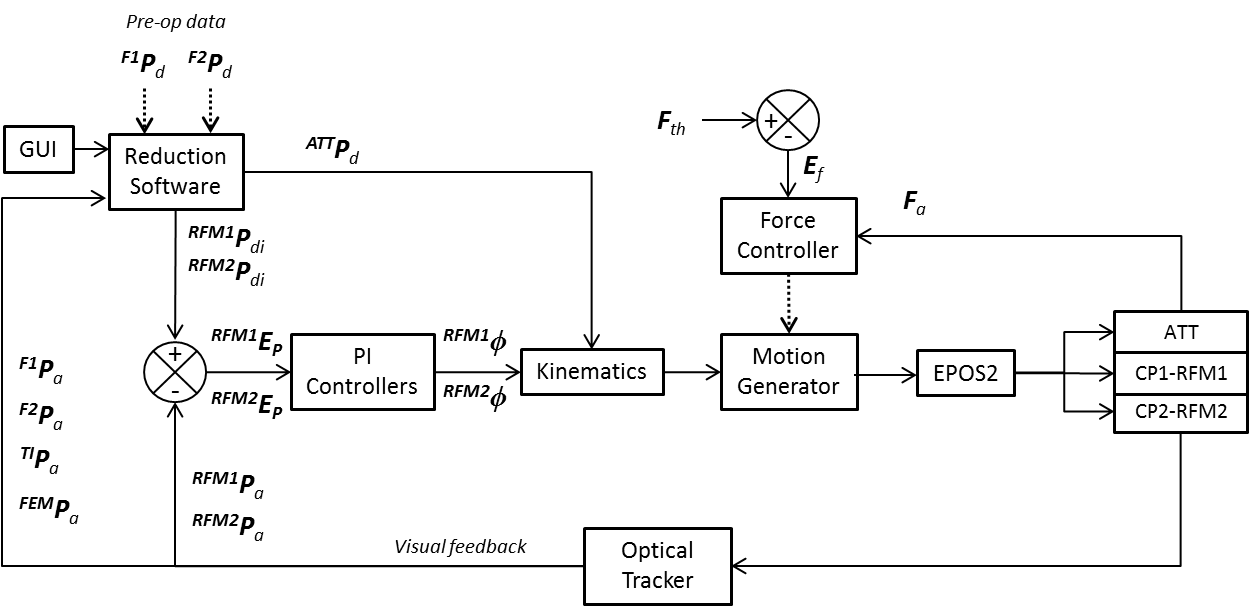

Supplement: Supplementary file 3 — Supplementary material 3 (PNG 35 kb) [file 10439_2017_1901_MOESM3_ESM.png]

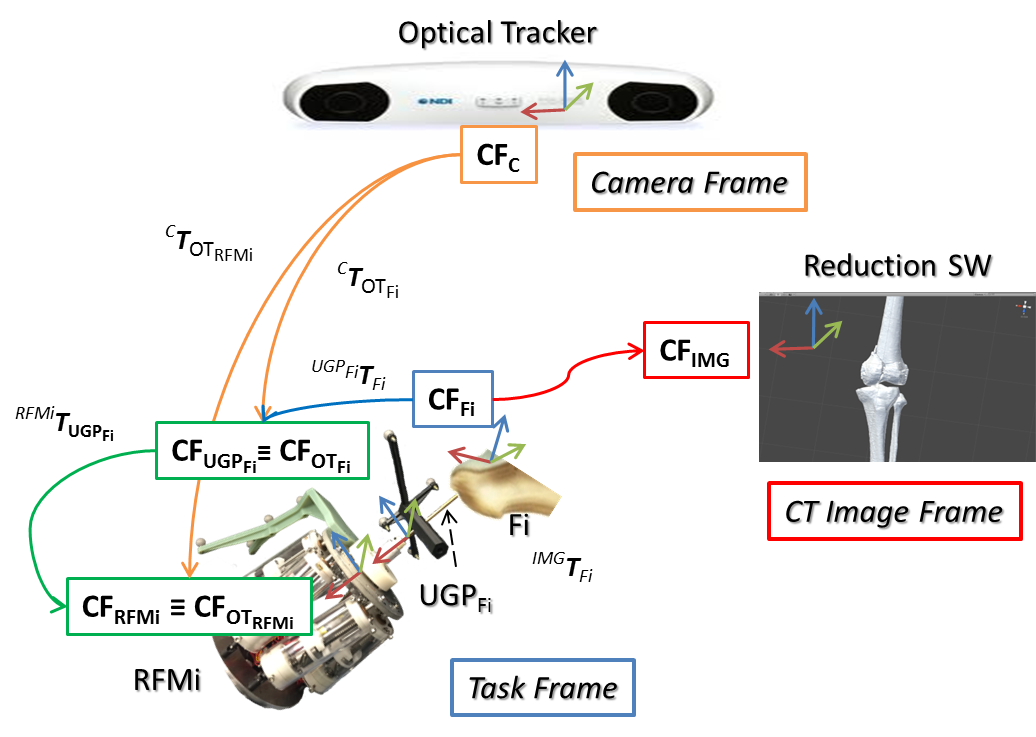

Supplement: Supplementary file 4 — Supplementary material 4 (PNG 211 kb) [file 10439_2017_1901_MOESM4_ESM.png]

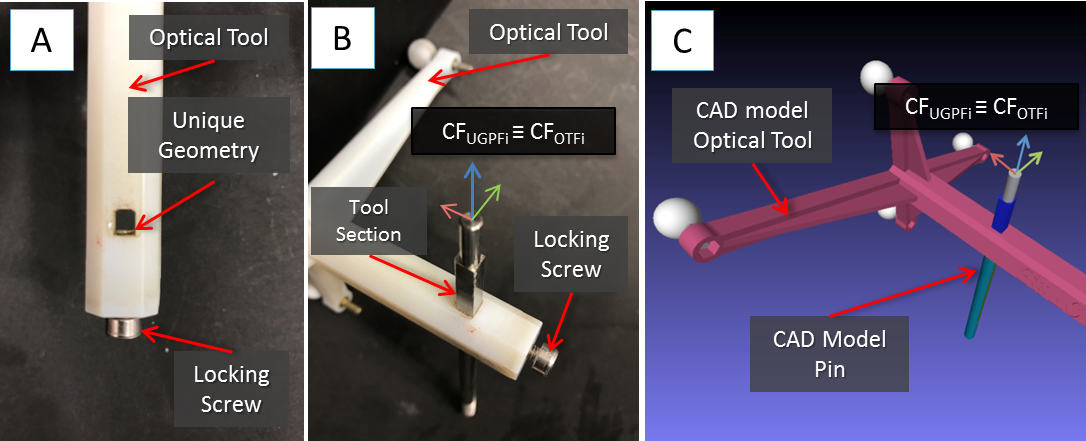

Supplement: Supplementary file 5 — Supplementary material 5 (PNG 451 kb) [file 10439_2017_1901_MOESM5_ESM.png]
